# Hypofractionated proton beam radiotherapy in patients with unresectable liver tumors: multi-institutional prospective results from the Proton Collaborative Group

**DOI:** 10.1186/s13014-020-01703-3

**Published:** 2020-11-04

**Authors:** Jacob S. Parzen, William Hartsell, John Chang, Smith Apisarnthanarax, Jason Molitoris, Michael Durci, Henry Tsai, James Urbanic, Jonathan Ashman, Carlos Vargas, Craig Stevens, Peyman Kabolizadeh

**Affiliations:** 1 Beaumont Proton Therapy Center, Royal Oak, MI USA; 2 Northwestern Medicine Chicago Proton Center, Warrenville, IL USA; 3 Oklahoma Proton Center, Oklahoma City, OK USA; 4 Seattle Cancer Care Alliance Proton Therapy Center, Seattle, WA USA; 5 Maryland Proton Treatment Center, Baltimore, MD USA; 6 Willis-Knighton Cancer Center, Shreveport, LA USA; 7 Princeton ProCure Proton Therapy Center, Kendall Park, NJ USA; 8 California Protons Therapy Center, San Diego, CA USA; 9 Mayo Clinic, Phoenix, AZ USA; 10 Department of Radiation Oncology, Oakland University William Beaumont School of Medicine, 3601 W Thirteen Mile Rd, Royal Oak, MI 48073 USA

**Keywords:** Proton therapy, Unresectable liver tumors, Hepatocellular carcinoma, Cholangiocarcinoma

## Abstract

**Background:**

Recent advances in radiotherapy techniques have allowed ablative doses to be safely delivered to inoperable liver tumors. In this setting, proton beam radiotherapy (PBT) provides the means to escalate radiation dose to the target volume while sparing the uninvolved liver. This study evaluated the safety and efficacy of hypofractionated PBT for liver tumors, predominantly hepatocellular carcinoma (HCC) and intrahepatic cholangiocarcinoma (ICC).

**Methods:**

We evaluated the prospective registry of the Proton Collaborative Group for patients undergoing definitive PBT for liver tumors. Demographic, clinicopathologic, toxicity, and dosimetry information were compiled.

**Results:**

To date, 63 patients have been treated at 9 institutions between 2013 and 2019. Thirty (48%) had HCC and 25 (40%) had ICC. The median dose and biological equivalent dose (BED) delivered was 58.05 GyE (range 32.5–75) and 80.5 GyE (range 53.6–100), respectively. The median mean liver BED was 13.9 GyE. Three (4.8%) patients experienced at least one grade ≥ 3 toxicity. With median follow-up of 5.1 months (range 0.1–40.8), the local control (LC) rate at 1 year was 91.2% for HCC and 90.9% for ICC. The 1-year LC was significantly higher (95.7%) for patients receiving BED greater than 75.2 GyE than for patients receiving BED of 75.2 GyE or lower (84.6%, *p* = 0.029). The overall survival rate at 1 year was 65.6% for HCC and 81.8% for ICC.

**Conclusions:**

Hypofractionated PBT results in excellent LC, sparing of the uninvolved liver, and low toxicity, even in the setting of dose-escalation. Higher dose correlates with improved LC, highlighting the importance of PBT especially in patients with recurrent or bulky disease.

## Introduction

The incidence of liver and intrahepatic bile duct tumors is increasing the most rapidly of any cancer in the United States [1]. Unfortunately, a minority of patients are eligible for curative surgical resection at presentation for either medical or anatomical reasons, leading to a dismal 18% 5-year overall survival rate [1]. Inoperable patients are often treated with other non-surgical local therapies, such as transarterial chemoembolization, transarterial radioembolization, and radiofrequency ablation. However, not all patients are candidates for these procedures, and eligible patients may not have a durable response to treatment. The role for potentially curative radiation therapy has been expanding considerably in this patient population [2]. With conventional fractionation, most patients experience local progression as the first site of failure after treatment [3]. However, technological advances in radiation treatment planning including four-dimensional respiratory motion management, image-guided radiation therapy, and proton therapy have made hypofractionated and ablative regimens safe and feasible [4–6].

Hepatocellular carcinoma (HCC) and intrahepatic cholangiocarcinoma (ICC) are commonly studied together due to shared clinical management. For HCC, multiple single-arm phase II trials have established the safety and efficacy of hypofractionated ablative radiation treatment [8–10]. Similarly, there have been prospective studies supporting this approach in ICC [5, 10]. A dose–response relationship for both clinical entities has been suggested [11, 12], but dose-escalation is ultimately limited by liver tolerance. The risk of radiation-induced liver disease (RILD) is influenced not only by the dose and volume of liver irradiated but also by the patient’s underlying liver disease.

Proton beam therapy (PBT) has the potential to improve dose conformity while sparing normal liver when compared to photon-based radiotherapy [13, 14]. In contrast to photon-based therapy, the proton Bragg peak yields a localized high-dose region in the tumor without exit dose. This dosimetric advantage spares a greater volume of uninvolved hepatic parenchyma and allows safer dose escalation in target volumes. Given the importance of proton therapy in treating liver tumors, the ongoing NRG GI-003 is focusing on evaluating the comparative efficacy of protons versus photons in patients with HCC. In this study, we used a multi-institutional prospective registry database to evaluate the safety and efficacy of ablative proton beam therapy for liver tumors, predominantly HCC and ICC.

## Methods and materials

REG001-09 (NCT01255748) is a prospective, multi-institutional registry of patients undergoing proton therapy at Proton Collaborative Group (PCG) institutions. Written informed consents were obtained from all patients before they were enrolled on the registry.

The registry trial was queried for patients undergoing definitive proton beam radiation therapy for liver tumors. Patients undergoing photon therapy were excluded. Patient, tumor characteristics, radiation treatment details, toxicity, and dosimetric information were all collected.

Patients underwent four-dimensional simulation with intravenous contrast. Immobilization method was per institutional discretion. All patients were treated with pencil beam scanning or passive scattering/uniform scanning. The relative biologic effectiveness (RBE) was set at 1.1 per institutional standard of all participating institutions. The dose unit Gy-equivalent (GyE) was proton dose in Gy multiplied by RBE. Fractionation schemes were at the discretion of participating institutions. Follow-up was institutional. Toxicity was graded using the National Cancer Institute Common Terminology Criteria for Adverse Events (CTCAE) version 4.0. We assumed an *α*/*β* = 10 for tumor effect and an *α*/*β* = 3 for radiation-induced liver toxicity. The linear-quadratic model was used for biological equivalent dose (BED) calculations.

Local control (LC), overall survival (OS), and progression-free survival (PFS) were calculated starting from the first day of radiation. The OS time of a patient still alive at the time of most recent follow-up was censored. A PFS event was defined as documented local or distant recurrence, or death, whichever was earlier, or otherwise was censored at last follow-up. OS and PFS rates were estimated using the Kaplan–Meier method and the differences between groups were compared with the log-rank test. The risk of local recurrence was estimated using the cumulative incidence function, treating death as a competing risk. Univariate analysis was performed using the Fine-Gray regression model [15]. Statistical analyses were performed using R version 3.6.2. Statistical significance was set to *P* < 0.05.

## Results

Sixty-three patients signed consent forms and were enrolled on the registry trial database. These patients were treated across 9 institutions between 2013 and 2019. The median number of patients treated per institution was 3 (range 1–17). Of the 63 patients, 30 had HCC, 25 had ICC, 4 had carcinoid tumors, 1 had spindle cell carcinoma, 1 had a liver metastasis, and 2 had unknown lesions. Patient characteristics are listed in Table 1. Child–Pugh status and the number of lesions present at the time of radiation were not collected on the registry. Thirty-two patients had smoking histories (51%), 25 did not (40%), and smoking status was unknown for 6 (10%).

**Table 1:** Patient characteristics

Characteristic	All (n = 63) % (no.) or median (range)	HCC (n = 30) % (no.) or median (range)	ICC (n = 25) % (no.) or median (range)
Age (years)	69 (29–89)	70.5 (34–89)	68 (29–87)
Sex			
Male	52% (33)	73% (22)	20% (5)
Female	48% (30)	27% (8)	80% (20)
Race/ethnicity			
White	75% (47)	73% (22)	76% (19)
Black	5% (3)	0% (0)	8% (2)
Asian	8% (5)	13% (4)	0% (0)
Hispanic	6% (4)	7% (2)	8% (2)
Unknown	6% (4)	7% (2)	8% (2)
ECOG			
0	41% (26)	43% (13)	28% (7)
1	43% (27)	33% (10)	64% (16)
2	8% (5)	17% (5)	0% (0)
3	2% (1)	3% (1)	0% (0)
Tumor dimension, cm	4.4 (0.6–17.0)	4.3 (1.2–9.4)	5.5 (0.6–17)
Previous therapy			
Surgery	13% (8)	13% (4)	12% (3)
TACE/TARE	30% (19)	53% (16)	8% (2)
RFA	6% (4)	13% (4)	0% (0)
Chemotherapy	30% (19)	10% (3)	60% (15)
Radiation therapy	10% (6)	10% (3)	12% (3)
None	40% (25)	37% (11)	32% (8)

*HCC* hepatocellular carcinoma, *ICC* intrahepatic cholangiocarcinoma, *TACE* transcatheter arterial chemoembolization, *TARE* transarterial radioembolization, *RFA* radiofrequency ablation

### Radiation dosing

Thirteen patients (21%) were treated with 5-fraction regimens, 46 (73%) were treated with 15-fraction regimens, and 4 (6%) were treated with 25-fraction regimens. Amongst patients treated with 5-fraction regimens, the median dose delivered was 40 GyE (range 32.5–50 GyE; BED range 53.6–100 GyE). Amongst the 15-fraction regimens, the median dose delivered was 58.05 GyE (range 45–67.5 GyE; BED range 58.5–97.9 GyE). Amongst the 25-fraction regimens, the median dose delivered was 71.1 GyE (range 60.1–75 GyE; BED range 74.5–97.5 GyE). The overall median biological equivalent dose (BED) was 80.5 GyE (range 53.6–100). The median mean dose received by the uninvolved liver (MLD) expressed in biological equivalent dose was 13.9 GyE (range 4.2–31.4). Fifty-two (83%) had no treatment breaks, and the remaining 11 (17%) had breaks in treatment but ultimately completed their treatment courses. Seven delays (64%) were attributed to the machine being down, four delays (27%) were secondary to personal patient issues not related to treatment toxicity, and one (9%) was due to the presence of bowel gas prohibiting safe delivery of treatment. One patient had 2 treatment breaks.

### Toxicity

Acute and chronic toxicities are presented in Table 2. The latencies to toxicity ranged from 0–38.3 months. Fifty-one (81%) of patients experienced at least one radiation-induced toxicity on the registry. The most common toxicities were fatigue (33/63, 52%), radiation dermatitis (21/63, 33%), anorexia (15/63, 24%), and nausea (13/63, 21%). Only three patients (4.8%) experienced at least one grade ≥ 3 toxicity. One HCC patient experienced grade 4 hyperbilirubinemia and grade 3 back pain. One ICC patient experienced grade 3 sinus bradycardia and another ICC patient experienced grade 3 abdominal pain. There were no grade 5 toxicities.

**Table 2:** Treatment-related toxicity

Toxicity			
CTCAE category	CTCAE term	Any grade % (No.)	Grade 3+ % (No.)
Blood/lymphatic system	Anemia	2% (1)	
	Other	2% (1)	
Cardiac	Sinus bradycardia	2% (1)	2% (1)
Gastrointestinal	Abdominal pain	11% (7)	2% (1)
	Bloating	3% (2)	
	Constipaton	14% (9)	
	Diarrhea	6% (4)	
	Dysphagia	6% (4)	
	Nausea	21% (13)	
	Stomach pain	3% (2)	
	Vomiting	2% (1)	
General	Fatigue	52% (33)	
	Pain	19% (12)	
Injury/procedural complications	Radiation Dermatitis	33% (21)	
Investigations	Hyperbilirubinemia	2% (1)	2% (1)
	Weight loss	3% (2)	
Metabolism	Anorexia	24% (15)	
Musculoskeletal	Back pain	6% (4)	2% (1)
	Bone pain	3% (2)	
Respiratory	Cough	2% (1)	
	Dyspnea	6% (4)	
Skin	Hyperpigmentation	3% (2)	

*CTCAE* common terminology criteria for adverse events (version 4)

### Disease-specific outcomes

**Fig. 1:**
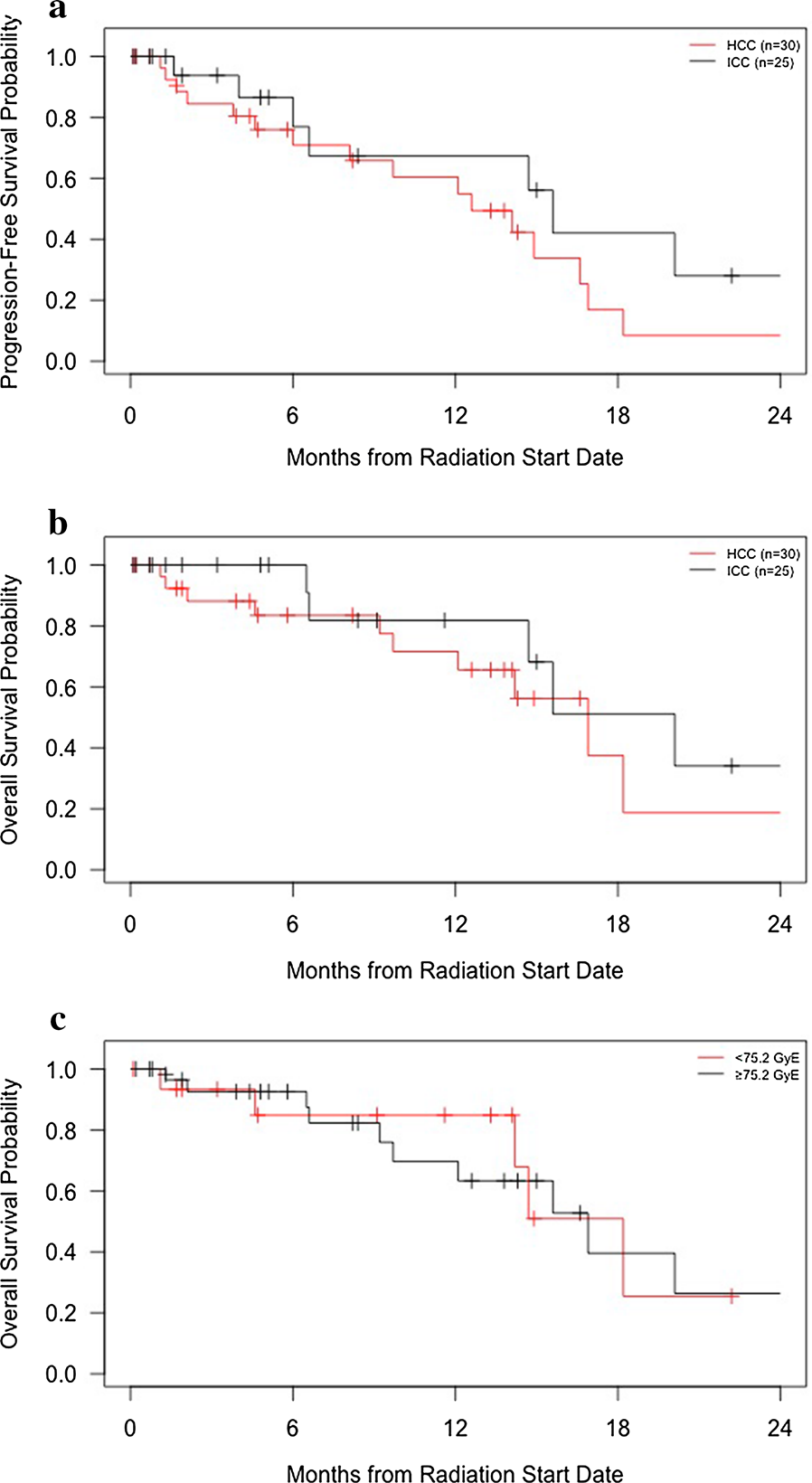
Progression-free survival and overall survival from the start of radiotherapy stratified by disease site (**a**, **b**) and overall survival stratified by dose (**c**)

Median follow-up was 5.1 months for the whole cohort (range 0.1–40.8 months). Patients with HCC had a median of 8.2 months follow-up, and their median PFS was 12.6 months (95% CI lower bound 8.1 months; upper bound not reached). The 1-year PFS rate was 60.3%. The median OS was 16.9 months (95% CI lower bound 12.1 months; upper bound not reached). The 1-year OS rate was 71.5% (Fig. 1a). Amongst ICC patients with median of 4.8 months follow-up, the median PFS was 15.6 months (95% CI lower bound 6.6 months; upper bound not reached). The 1-year PFS rate was 67.3%. The median OS was 20.1 months (95% CI lower bound 14.7 months; upper bound not reached). The 1-year OS rate was 81.8%. There was no difference in OS (*p* = 0.2) or KPS (*p* = 0.1) between patients with HCC and ICC (Fig. 1a, b). For patients with HCC and ICC, the median OS was 18.2 months (95% CI lower bound 14.2 months; upper bound not reached) for those patients receiving < 75.2 GyE BED and 16.9 months (95% CI lower bound 12.1 months; upper bound not reached) for those patients receiving ≥ 75.2 GyE BED (*p* = 0.9) (Fig. 1c).

**Fig. 2:**
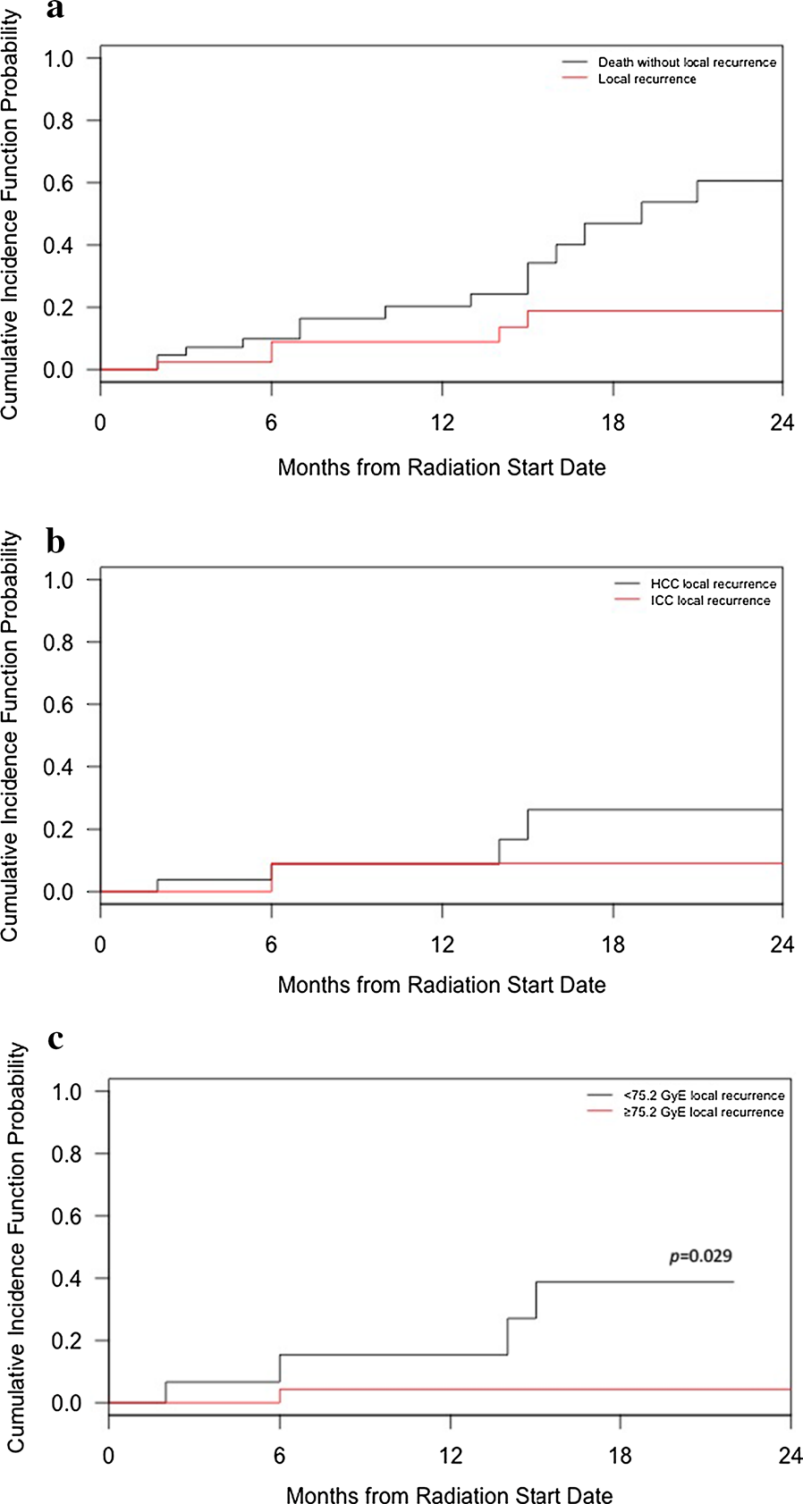
Cumulative incidence function for local recurrence for all patients (**a**); by disease type (**b**); and by radiation dose (**c**)

Five patients (four HCC and one ICC) experienced local failure within 2 years of follow-up. For patients with HCC and ICC, the 1-year LC rate was 91.1% (95% CI 78.4–97.8%) and the 2-year LC rate was 81.1% (95% CI 63.2–93.8%) (Fig. 2a). Hence, evaluating the death without local recurrence as a competing factor confirms excellent local control with escalated dose radiotherapy. The 1-year LC rate was 91.2% for HCC and 90.9% for ICC (Fig. 2b). On cumulative incidence risk analysis, LC was significantly higher for those patients receiving ≥ 75.2 GyE BED than for those patients receiving < 75.2 GyE BED (1-year LC 95.7% versus 84.6%, *p* = 0.029, Fig. 2c). However, there was no difference in overall survival (*p* = 0.483). Factors associated with improved local control are displayed in Table 3. There was a trend towards a statistically significant association with BED (as a continuous variable) and local control (*p* = 0.057). Multivariate analysis was not completed due to the low number of events.

**Table 3:** Univariate analysis for local control

Variable	Level	HR	95% CI	*P*
Age		1.02	0.97–1.08	0.35
ECOG	1–3 v 0	2.50	0.29–21.80	0.41
Prior treatment	Yes vs. No	0.53	0.10–2.81	0.45
Tumor size (cm)		0.87	0.65–1.17	0.36
Fractionation	15/25 vs. 5	0.39	0.07–1.99	0.26
BED, GyE ±		0.97	0.93–1.00	0.057

*ECOG* Eastern Cooperative Oncology Group performance status, *BED* biological equivalent dose, *HR* hazard ratio, *GyE* Gy equivalent

± BED considered as a continuous variable. The HR represents the effect of an increase of 1 GyE

Patterns of failure and death for HCC and ICC patients are displayed in Table 4. Amongst HCC and ICC patients, 59% and 78% of patients were alive at last follow-up or had expired from an unrelated cause to their cancer, respectively. All isolated local failures were without distant failure in both groups.

**Table 4:** Patterns of failure

PFS Status	All (n = 63) % (No.)	HCC (n = 30) % (No.)	ICC (n = 25) % (No.)
Alive, no progression	57% (36)	43% (13)	68% (17)
Distant metastases	17% (11)	23% (7)	12% (3)
Local failure and distant metatases	0% (0)	0% (0)	0% (0)
Isolated local failure	8% (5)	13% (4)	4% (1)
Dead of this disease, no progression	3% (2)	3% (1)	4% (1)
Dead of other causes, no progression	14% (9)	16% (5)	10% (3)

*PFS* progression-free survival, *HCC* hepatocellular carcinoma, *ICC* intrahepatic cholangiocarcinoma

## Discussion

In this prospective registry of patients undergoing definitive proton therapy for liver tumors, we demonstrate excellent local control with low rates of toxicity. Our rates of local control are comparable to hypofractionated historical series [16–18]. In the absence of phase III data, this adds to the growing body of literature validating this approach and making a compelling case that it should be placed alongside other liver-directed therapies as standard-of-care in patients who are not operable candidates.

By virtue of the Bragg peak, proton radiotherapy offers a distinct dosimetric advantage when compared to photon radiotherapy. An earlier study through Loma Linda, treated 34 patients with HCC to 63 GyE in 15 fractions, reporting a 2-year LC of 75% [19]. Importantly, there were no cases of RILD. Other studies have corroborated low rates of RILD in patients receiving proton therapy when compared to photon therapy [20, 21], allowing for treatment of large lesions beyond 6 cm without any significant toxcities [22]. A recent retrospective comparison of proton and photon ablative radiotherapy in patients with unresectable HCC demonstrated an overall survival benefit in patients receiving proton therapy despite no difference in locoregional control [23]. The survival benefit was rather attributed to an increased risk of non-classic RILD, defined as an increase in the baseline Child–Pugh score of ≥ 2 at 3 months posttreatment, in patients receiving photon-based radiotherapy. Through elimination of the low-dose bath associated with photon therapy, there is relative protection of normal liver parenchyma while still delivering ablative doses to the area of interest, which may have improved clinical outcomes.

Our study builds upon previous prospective experiences treating inoperable HCC and ICC. Hong et al. conducted a single-arm phase II study treating patients with HCC and ICC to 67.5 GyE in 15 fractions for peripheral tumors and 58.05 Gy in 15 fractions for central tumors with proton beam radiotherapy at three institutions [10]. With median follow-up of 19.5 months, they reported 2-year LC of 94.8% for HCC and 94.1% for ICC. The rate of grade 3 toxicity was 4.8%. Though all patients on that trial were treated with 15 fractions, the median BED of 80.4 GyE was nearly identical to our experience. Importantly, the 5 fraction patients in our experience do not fare worse from a local control standpoint, supporting extreme hypofractionation as long as sufficient BED is administered. In another similar experience at the Princess Margaret Hospital, 102 patients with HCC were treated on a phase I/II trial from 24 to 54 Gy in 6 fractions with photon radiotherapy [8]. They reported a 1-year LC rate of 87%. However, the rate of grade ≥ 3 toxicity was 30%, including 7 cases of grade 5 toxicity.

The differences in toxicities are likely multifactorial. First, the average sum of all liver lesions on the Princess Margaret study was 9.9 cm, compared to 5.8 cm on the Hong et al. study and 4.4 cm on our study. Caution must be taken when treating very large tumors with extreme hypofractionation. The second and more intriguing possibility is that proton therapy improves toxicity profiles by limiting dose to the uninvolved liver. Using the linear-quadratic model, the median MLD in BED was 13.9 GyE amongst our patients, versus 27.4 GyE on the Hong et al. trial and 30.0 GyE in the Princess Margaret experience. We acknowledge that the tumors on this registry were smaller than the other studies, which would partially account for the difference. It is also likely that the liver-sparing afforded by proton therapy results in lower dose to normal liver which in turn lowers the deleterious effects on liver reserve. The low rates of grade ≥ 3 toxicity on both the Hong et al. and current study corroborate the importance of liver sparing. As previously mentioned, this is important because lowering the rates of treatment-related liver decompensation may improve clinical outcomes [24]. While the Princess Margaret experience did also raise concerns about extreme hypofractionation from a toxicity standpoint, this was not found in our patients undergoing a 5-fraction schedule, suggesting this approach is safe and feasible in appropriately-selected patients.

The current study also corroborates previous findings that there exists a dose–response relationship with respect to tumor control. This did not translate into an overall survival benefit most likely due to short follow-up, and possibly competing risks of new lesions, metastatic disease and liver failure. This is still of critical importance given the first site of progression after radiation therapy is predominantly local [3]. The early University of Michigan experience, on which patients with primary liver tumors or metastases were treated to a median dose of 58.5 Gy in 1.5 Gy twice daily fractions, demonstrated a median survival of 16.4 months in patients treated with at least 70 Gy (BED 80.5 Gy), compared to 11.6 months in patients treated with lower dose [25]. Early data from the Hong et al. phase II trial also suggested a local control and survival benefit with increasing BED (per NRG-GI 003 protocol). More recently, a retrospective experience with inoperative ICC, on which patients were treated to a median BED of 80.5 Gy with both conventional fractionation and hypofractionation, the 3-year LC for patients receiving BED greater than 80.5 Gy was 73% versus 38% for those receiving less, and BED as a continuous variable significantly affected both LC and OS [12]. An important criticism of that study is that a large subset of patients were treated with 50.4 Gy in 28 fractions, which is not a radio-ablative regimen. Our study establishes a dose cut-off that is even lower at 75.2 GyE, but there was only a trend towards improvement in LC when BED was evaluated as a continuous variable. The lower threshold established herein could be explained in part by the uncertainly regarding an assumed RBE factor of 1.1 [26] as is the convention in the participating institutions. However, other studies evaluating proton therapy have established BED cut-offs as high as 90 GyE [11].

Despite the above, proton therapy as a treatment modality remains somewhat controversial given its cost and lack of level I evidence for overall survival, thus prompting the ongoing phase III NRG trial randomizing patients to protons versus photons in patients with HCC [6]. Strengths of the current study include its prospective registry nature and multidisciplinary contributions, affording reasonable sample size for this relatively rare disease entity. Conversely, a notable weakness is the lack of HCC/ICC-specific data included in the registry, including but not limited to Child–Pugh scoring, the presence of tumor vascular thrombosis, the presence of multiple tumors, and more detailed plan information such as volume of liver irradiated. In addition, the relatively limited follow-up time in some patients likely biased patterns of failure results. In addition, due to the disparity in follow-up between the HCC and ICC patients, any differences between the groups reported herein should be interpreted with extreme caution.

In conclusion, hypofractionated ablative proton therapy is safe and efficacious in the treatment of primary liver tumors. We have reaffirmed a dose–response relationship and highlighted the importance of dose-escalation in local control. Inasmuch as it is possible while respecting critical dose constraints, clinicians should attempt to deliver a biological equivalent dose of at least 75.2 GyE to unresectable liver tumors when the intent is ablation. The marginal clinical benefit of liver-sparing proton therapy is currently being investigated in a phase III trial.

## Data Availability

The datasets generated and analyzed during the current study are not publically available but are available from the corresponding author on reasonable request.
